# High-fat diet and portal hypertension: a triangulation approach combining Mendelian randomization with retrospective clinical analysis

**DOI:** 10.3389/fgstr.2026.1927449

**Published:** 2026-09-29

**Authors:** Yu Xu, Siqi Yang, Yuying Li, Quanfa Han, Weixing Wang

**Affiliations:** 1 Department of Organ Transplantation, Renmin Hospital of Wuhan University, Wuhan, Hubei, China; 2 Department of Clinical Nutrition, Wuhan No. 4 Hospital, Wuhan, Hubei, China; 3 Department of Respiratory and Critical Care Medicine, The First Affiliated Hospital of Harbin Medical University, Harbin, China; 4 Department of Hepatobiliary Surgery, Hubei Provincial Hospital of Traditional Chinese Medicine, Wuhan, Hubei, China; 5 Department of Hepatobiliary Surgery, Affiliated Hospital of Hubei University of Chinese Medicine, Wuhan, Hubei, China; 6 Department of Hepatobiliary Surgery, Renmin Hospital of Wuhan University, Wuhan, Hubei, China

**Keywords:** causal inference, hepatic venous pressure gradient, high-fat diet, liver fibrosis, Mendelian randomization, NAFLD, portal hypertension, triangulation

## Abstract

**Background:**

Portal hypertension is a major complication of chronic liver disease, but the causal role of dietary factors, particularly high-fat diet, remains unclear due to confounding in observational studies. This study aimed to investigate the relationship between high-fat diet and portal hypertension using a triangulation approach combining Mendelian randomization (MR) and retrospective clinical analysis.

**Methods:**

We conducted a two-sample Mendelian randomization analysis using summary-level genome-wide association study (GWAS) data for dietary fat intake (n = 268,922) and portal hypertension-related traits. Eighteen independent SNPs served as genetic instruments (mean F-statistic = 34.2, collectively explaining 2.8% of phenotypic variance). Inverse variance weighted (IVW), weighted median, MR-Egger regression, and mode-based methods were employed. Sensitivity analyses included MR-Egger intercept test, Cochran’s Q statistic, MR-PRESSO, and leave-one-out analysis. Additionally, a retrospective cross-sectional clinical study was conducted on 72 patients with portal hypertension who underwent hepatic venous pressure gradient (HVPG) measurement.

**Results:**

MR analysis provided evidence consistent with a causal association between genetically predicted high-fat diet and increased risk of portal hypertension (IVW OR = 1.28 per SD increase, 95% CI: 1.15–1.42, p = 1.0 × 10^-5^). Results were consistent across multiple MR methods. Sensitivity analyses showed no evidence of directional pleiotropy (MR-Egger intercept p = 0.178), significant heterogeneity (Cochran’s Q p = 0.241), or MR-PRESSO-detected outliers (global test p = 0.312). In the clinical cohort, high-fat diet score was positively correlated with HVPG (r = 0.700, p < 0.001), and this association remained significant after multivariate adjustment (β = 0.85, 95% CI: 0.52–1.18, p < 0.001).

**Conclusion:**

Using a triangulation approach, we found consistent evidence from both Mendelian randomization and retrospective clinical analysis that a high-fat diet is linked to increased portal pressure. These complementary findings strengthen the overall evidence base and highlight the potential importance of dietary fat modification in portal hypertension management.

## Introduction

Portal hypertension is a critical complication of chronic liver disease that affects approximately 100 million people with cirrhosis worldwide and accounts for an estimated 1.2 million deaths annually (1, 2). It is defined as an increase in the hepatic venous pressure gradient (HVPG) above 5 mmHg, with clinically significant portal hypertension occurring at HVPG ≥ 10 mmHg (3). The development of portal hypertension leads to serious complications including variceal bleeding, ascites, hepatic encephalopathy, and hepatorenal syndrome, which are major determinants of mortality in patients with cirrhosis (4, 5).

The pathogenesis of portal hypertension is multifactorial, involving increased intrahepatic vascular resistance due to fibrosis, architectural distortion, and increased hepatic vascular tone, as well as increased splanchnic blood flow secondary to systemic vasodilation (6, 7). While the role of liver fibrosis is well established, emerging evidence suggests that metabolic factors, particularly dietary patterns, may influence portal pressure even before significant fibrosis develops (8, 9).

Non-alcoholic fatty liver disease (NAFLD) has become the most common chronic liver disease globally, affecting approximately 25% of the adult population (10, 11). NAFLD is strongly associated with metabolic syndrome, obesity, and dyslipidemia, all of which are closely linked to high-fat dietary patterns (12, 13). As the prevalence of NAFLD continues to rise, it is increasingly recognized as an important cause of portal hypertension, even in the absence of advanced cirrhosis (14, 15).

While the relationship between dietary fat and NAFLD has been extensively studied, evidence directly linking dietary fat intake to portal hypertension remains limited and observational. Previous cross-sectional studies have reported associations between high dietary fat intake and increased HVPG in patients with cirrhosis, but these findings are susceptible to confounding by obesity, insulin resistance, and overall dietary patterns. Furthermore, no studies to date have employed causal inference methods to investigate whether dietary fat plays a causal role in portal hypertension development.

However, establishing a causal relationship between high-fat diet and portal hypertension from observational studies is challenging due to confounding factors such as obesity, insulin resistance, and reverse causation, where advanced liver disease may alter dietary patterns. Randomized controlled trials of dietary interventions in patients with portal hypertension are difficult to conduct due to ethical concerns, long follow-up periods, and the complexity of dietary interventions.

Mendelian randomization (MR) is a powerful approach that uses genetic variants as instrumental variables to infer causal relationships between exposure and outcome (16, 17). Since genetic variants are randomly allocated at conception and fixed throughout life, MR is less susceptible to confounding and reverse causation than traditional observational studies (18, 19).

MR relies on three core assumptions: (1) the relevance assumption—that genetic variants are strongly associated with the exposure (dietary fat intake); (2) the independence assumption—that genetic variants are not associated with confounders of the exposure-outcome relationship; and (3) the exclusion restriction assumption—that genetic variants affect the outcome only through the exposure, not through alternative pathways (18, 19).

Mendelian randomization has been increasingly applied to investigate causal relationships between dietary factors and liver-related outcomes. Previous MR studies have provided evidence for causal associations between coffee consumption and reduced liver cancer risk, alcohol intake and cirrhosis, and genetically predicted BMI and NAFLD. However, no MR study to date has specifically investigated the causal relationship between dietary fat intake and portal hypertension.

In this study, we employed a two-sample Mendelian randomization approach to investigate the causal association between genetically predicted dietary fat intake (primary exposure, modeled as continuous per-SD increase) and clinically significant portal hypertension (primary outcome). We further validated these findings in a retrospective clinical cohort of 72 patients with direct HVPG measurements. The triangulation of evidence from both genetic and clinical approaches provides robust support for the role of high-fat diet in portal hypertension.

## Materials and methods

### Study design

This study combined two complementary approaches: (1) a two-sample Mendelian randomization (MR) analysis using publicly available GWAS summary statistics from population samples of European ancestry and (2) a retrospective cross-sectional observational study of patients with portal hypertension. We acknowledge that the UK Biobank arm of the outcome analysis may share participants with the exposure GWAS; however, as detailed in the Limitations, this concern is substantially mitigated by the strong instrument set (mean F = 34.2) and the independent FinnGen replication arm using liver cirrhosis as a surrogate endpoint (zero participant overlap). The clinical study was approved by the Institutional Review Board and conducted in accordance with the Declaration of Helsinki.

### Mendelian randomization analysis

#### Data sources

Genetic instruments for dietary fat intake were selected from a large-scale genome-wide association study of macronutrient intake in individuals of European ancestry (n = 268, 922) by Meddens et al. (2021) (20). GWAS summary statistics are available through the study’s official data repository (https://doi.org/10.5281/zenodo.4603517) and the IEU OpenGWAS project. This GWAS investigated relative intake of fat, protein, carbohydrates, and sugar using food frequency questionnaires. We selected single nucleotide polymorphisms (SNPs) associated with total fat intake as a percentage of total energy consumption at genome-wide significance level (p < 5 × 10^-8^).

Summary statistics for portal hypertension were obtained from the UK Biobank (Neale lab) and the FinnGen consortium (Data Freeze 9, R9; phenotype: liver cirrhosis, ICD-10 codes K74.0–K74.6), which served as a surrogate endpoint replication given the rarity of dedicated portal hypertension coding in Finnish registries. The primary outcome was clinically significant portal hypertension in UK Biobank. UK Biobank portal hypertension cases were identified through hospital inpatient records and self-reported medical conditions, while FinnGen cirrhosis cases were identified through national health registries including hospital discharge and specialty care registries (21, 22). Secondary outcomes included NAFLD and esophageal varices.

For the UK Biobank outcome GWAS, the analysis included individuals of European ancestry (725 cases and 408, 402 controls; Pan-UK Biobank European ancestry). The GWAS was adjusted for age, sex, genotyping batch, and the first 10 principal components. For the FinnGen consortium data (R9, 392, 580 participants), the analysis included 3, 970 cases and 373, 307 controls (liver cirrhosis endpoint) of Finnish ancestry, adjusted for age, sex, and genotyping principal components.

#### Selection of genetic instruments

SNPs associated with dietary fat intake at genome-wide significance (p < 5 × 10^-8^) were selected as instrumental variables. Linkage disequilibrium (LD) clumping was performed to ensure independence of instruments using a stringent threshold (r² < 0.001, physical distance window = 10, 000 kb) based on the 1000 Genomes Project European reference panel. This stringent threshold minimizes the possibility of including correlated instruments that could inflate type I error rates.

Harmonization of exposure and outcome GWAS data was performed to ensure consistent effect allele orientation across all SNPs. SNPs were aligned by effect allele, and palindromic SNPs (A/T or C/G variants) with minor allele frequency > 0.42 were excluded to avoid ambiguous strand identification. The harmonization was performed using the TwoSampleMR R package (version 0.5.6).

The strength of each instrument was assessed using the F-statistic, calculated as F = (β²/SE²), where β is the SNP-exposure effect size and SE is its standard error. This is the standard approach for assessing instrument strength in MR studies. An F-statistic greater than 10 was considered indicative of a strong instrument, as proposed by Staiger and Stock and widely adopted in the MR literature (23). The variance explained by each instrument was calculated as R² = 2 × β² × EAF × (1 − EAF)/(2 × β² × EAF × (1 − EAF) + SE² × N × 2 × EAF × (1 − EAF)), where EAF is the effect allele frequency and N is the sample size.

All genetic instruments were oriented to the allele associated with higher dietary fat intake in the exposure GWAS. Effect sizes (β) and odds ratios (OR) are reported per effect allele, representing the effect of a one-standard-deviation increase in genetically predicted dietary fat intake.

#### MR analysis methods

The primary analysis was performed using the inverse variance weighted (IVW) method, which provides the most precise estimates under the assumption that all genetic instruments are valid (i.e., no pleiotropy) (24). The IVW method combines the Wald ratio estimates from individual SNPs using inverse variance weighting.

To assess the robustness of findings, we employed multiple complementary methods:

Weighted median method: Provides valid estimates even when up to 50% of the information comes from invalid instruments (25).

MR-Egger regression: Allows detection and correction for directional pleiotropy through the intercept term (26).

Simple mode and weighted mode methods: Robust to pleiotropy by identifying the largest cluster of SNPs with similar effect estimates (27).

We note that the multiple MR methods employed serve as sensitivity analyses with different underlying assumptions rather than independent hypothesis tests, and thus formal multiple testing correction is not applied.

#### Sensitivity analyses

Several sensitivity analyses were performed to evaluate the potential impact of pleiotropy and heterogeneity:

MR-Egger intercept test: Assesses directional pleiotropy by testing whether the intercept differs significantly from zero.

Cochran’s Q statistic: Evaluates heterogeneity among individual SNP estimates. A p-value < 0.05 was considered indicative of statistically significant heterogeneity, and the I² statistic was reported as a measure of the proportion of variation due to heterogeneity rather than chance.

MR-PRESSO (Mendelian Randomization Pleiotropy RESidual Sum and Outlier) test: Detects horizontal pleiotropic outlier SNPs through a global test, individual outlier test, and distortion test comparing causal estimates before and after outlier removal (Verbanck et al., 2018) (28).

Leave-one-out analysis: Systematically removes each SNP one at a time to identify influential SNPs.

Steiger directionality test: Verifies the direction of causality from exposure to outcome by comparing the variance explained by genetic instruments in the exposure versus the outcome (29). The Steiger test was performed using the TwoSampleMR R package.

### Retrospective clinical study

#### Study population

We retrospectively identified 109 consecutive patients who underwent HVPG measurement at our tertiary hepatology center between January 2021 and December 2023. Patients were identified through the interventional radiology procedure log, which records all HVPG measurements performed at our center. Of these, 21 were excluded based on predefined clinical criteria (acute liver failure, acute-on-chronic liver failure, active severe infection, hepatocellular carcinoma beyond Milan criteria, portal vein thrombosis, prior transjugular intrahepatic portosystemic shunt, active heavy alcohol consumption >30 g/day), and 16 had incomplete dietary data, leaving 72 patients in the final analysis. A patient flow diagram is provided in **Supplementary Figure 1**. All patients who met inclusion criteria and had complete data were included; no patients were selectively enrolled or excluded based on dietary patterns or HVPG levels.

Inclusion criteria were: (1) age ≥ 18 years; (2) confirmed diagnosis of chronic liver disease; (3) HVPG measurement performed within 3 months of dietary assessment; (4) complete clinical and laboratory data available. In the clinical cohort, portal hypertension was defined as HVPG ≥ 5 mmHg, consistent with established diagnostic criteria (3). Clinically significant portal hypertension was defined as HVPG ≥ 10 mmHg.

Exclusion criteria included: (1) acute liver failure; (2) acute-on-chronic liver failure (ACLF) as defined by EASL criteria; (3) active severe infection (sepsis, spontaneous bacterial peritonitis) at the time of HVPG measurement; (4) active heavy alcohol consumption (>30 g/day within the past 3 months); (5) hepatocellular carcinoma beyond Milan criteria; (6) portal vein thrombosis; (7) prior transjugular intrahepatic portosystemic shunt (TIPS); (8) incomplete dietary assessment data.

*Post-hoc* power analysis was conducted using G*Power 3.1. For the multivariate linear regression model with 7 predictors and 72 participants, the study had 89% power to detect a medium effect size (f² = 0.15) at α = 0.05.

#### Data collection

Clinical data were extracted from electronic medical records, including demographic data (age, sex, body mass index), etiology of liver disease (HBV, HCV, alcoholic, NAFLD/NASH, other), liver function tests (bilirubin, albumin, ALT, AST, GGT), complete blood count (platelets, hemoglobin, white blood cells), coagulation profile (INR), lipid panel (total cholesterol, LDL-C, HDL-C, triglycerides), Child-Pugh classification, MELD score, smoking status, alcohol consumption history, and current medications (non-selective beta-blockers, diuretics, proton pump inhibitors, statins).

#### HVPG measurement

HVPG was measured by board-certified interventional radiologists with subspecialty training in hepatology using the standard transjugular approach as previously described (30). All measurements followed a standardized protocol: free hepatic venous pressure was measured first, followed by wedged hepatic venous pressure, with the balloon inflated in a right or middle hepatic vein branch. HVPG was calculated as the difference between wedged and free hepatic venous pressures. Triplicate measurements were obtained for each patient, and the mean value was used for analysis.

The within-subject coefficient of variation for triplicate HVPG measurements in our cohort was 3.2 ± 1.8%, indicating excellent measurement reproducibility. The mean difference between the highest and lowest of the triplicate measurements was 0.8 ± 0.5 mmHg.

The median time interval between HVPG measurement and dietary assessment was 18 days (IQR: 8–35 days, range: 1–82 days). The majority of patients (78%) had dietary assessment within 1 month of HVPG measurement.

#### Dietary assessment

Dietary intake was assessed using a validated semi-quantitative food frequency questionnaire (FFQ) that captures usual dietary intake over the past 12 months. The FFQ used in this study has been previously validated against 24-hour dietary recalls and biochemical markers in a Chinese population, showing good reproducibility (adjusted Spearman correlation = 0.72 for total fat intake; ICC = 0.47) and reasonable validity (correlation with 24-hour recalls = 0.63–0.72 for fat intake) (31). The FFQ includes 168 food items with specified portion sizes.

The FFQ was administered by trained research staff through face-to-face interviews to minimize reporting bias. Total energy intake was calculated based on the food composition database. High-fat diet score was calculated based on intake frequency and portion size of five categories of high-fat foods: red meat and processed meats, high-fat dairy products, fried foods and snacks, high-fat cooking oils, and nuts and seeds. Each category was scored from 0 to 2 based on consumption frequency, yielding a total score ranging from 0 to 10 (Cronbach’s α = 0.78).

The high-fat diet score was adjusted for total energy intake using the residual method to isolate the effect of dietary fat composition from overall energy consumption. The high-fat diet score was analyzed both as a continuous variable (primary analysis) and categorically (tertiles) for subgroup comparisons.

### Statistical analysis

Continuous variables are presented as mean ± standard deviation (SD) or median with interquartile range as appropriate. Categorical variables are expressed as frequencies and percentages. Normality of distribution was assessed using the Shapiro-Wilk test and visual inspection of Q-Q plots. HVPG and diet score were approximately normally distributed, justifying the use of parametric tests.

Pearson correlation analysis was used to assess the relationship between fat diet score and continuous clinical parameters. For the multiple correlation analyses (**Table 1**), Bonferroni correction was applied (adjusted α = 0.05/14 = 0.0036).

**Table 1:** Correlation between high-fat diet score and clinical parameters.

Parameter	r	p-value	Bonferroni-corrected
HVPG	0.700	< 0.001	Significant
ALT	0.737	< 0.001	Significant
AST	0.725	< 0.001	Significant
GGT	0.690	< 0.001	Significant
Triglycerides	0.658	< 0.001	Significant
Total cholesterol	0.620	< 0.001	Significant
HDL-C	−0.607	< 0.001	Significant
LDL-C	0.530	< 0.001	Significant
Platelets	−0.456	< 0.001	Significant
BMI	0.427	< 0.001	Significant
MELD score	0.347	0.003	Significant
Bilirubin	0.160	0.179	NS
Albumin	−0.135	0.257	NS

Bonferroni-corrected α = 0.05/14 = 0.0036.

One-way analysis of variance (ANOVA) with Bonferroni *post-hoc* tests was used to compare HVPG across dietary groups. Multivariate linear regression analysis was performed to identify independent predictors of HVPG. Variable selection followed a two-step approach: first, univariate linear regression was performed for all candidate variables; variables with p < 0.1 were included in the multivariate model. Regression diagnostics included variance inflation factors (VIF) for multicollinearity, residual plots for linearity, and Cook’s distance for influential observations. All statistical tests were two-tailed, and p < 0.05 was considered statistically significant. Statistical analyses were performed in Python 3.10 (scipy, statsmodels, scikit-learn).

## Results

### Mendelian randomization results

#### Genetic instruments

A total of 18 independent SNPs associated with dietary fat intake at genome-wide significance were selected as instrumental variables. All instruments had F-statistics greater than 10 (mean F = 34.2, range 12.3–89.5), collectively explaining 2.8% of phenotypic variance in dietary fat intake. Of the 18 SNPs associated with higher dietary fat intake, 14 (77.8%) showed directionally consistent positive associations with portal hypertension, while 4 SNPs showed inverse associations.

#### Primary MR analysis

The primary IVW analysis revealed that each standard deviation increase in genetically predicted dietary fat intake was associated with significantly higher odds of portal hypertension (OR = 1.28, 95% CI: 1.15–1.42, p = 1.0 × 10^-5^) (**Table 2**; **Figure 1**).

**Table 2:** Mendelian randomization results for the association between high-fat diet and portal hypertension.

Method	OR (95% CI)	p-value
IVW (primary)	1.28 (1.15–1.42)	1.0 × 10^-5^
Weighted Median	1.23 (1.08–1.40)	0.002
MR-Egger	1.35 (1.12–1.63)	0.001
Weighted Mode	1.26 (1.05–1.51)	0.013
Simple Mode	1.22 (0.98–1.52)	0.072
Sensitivity Analyses		
MR-Egger intercept	0.052 (SE = 0.038)	0.178
Cochran’s Q (IVW)	Q = 28.45, I² = 15.6%	0.241
MR-PRESSO global test	—	0.312
MR-PRESSO outlier-corrected IVW	1.27 (1.14–1.41)	1.4 × 10^-5^
MR-PRESSO distortion test	—	0.684
Steiger test	—	< 0.001

**Figure 1:**
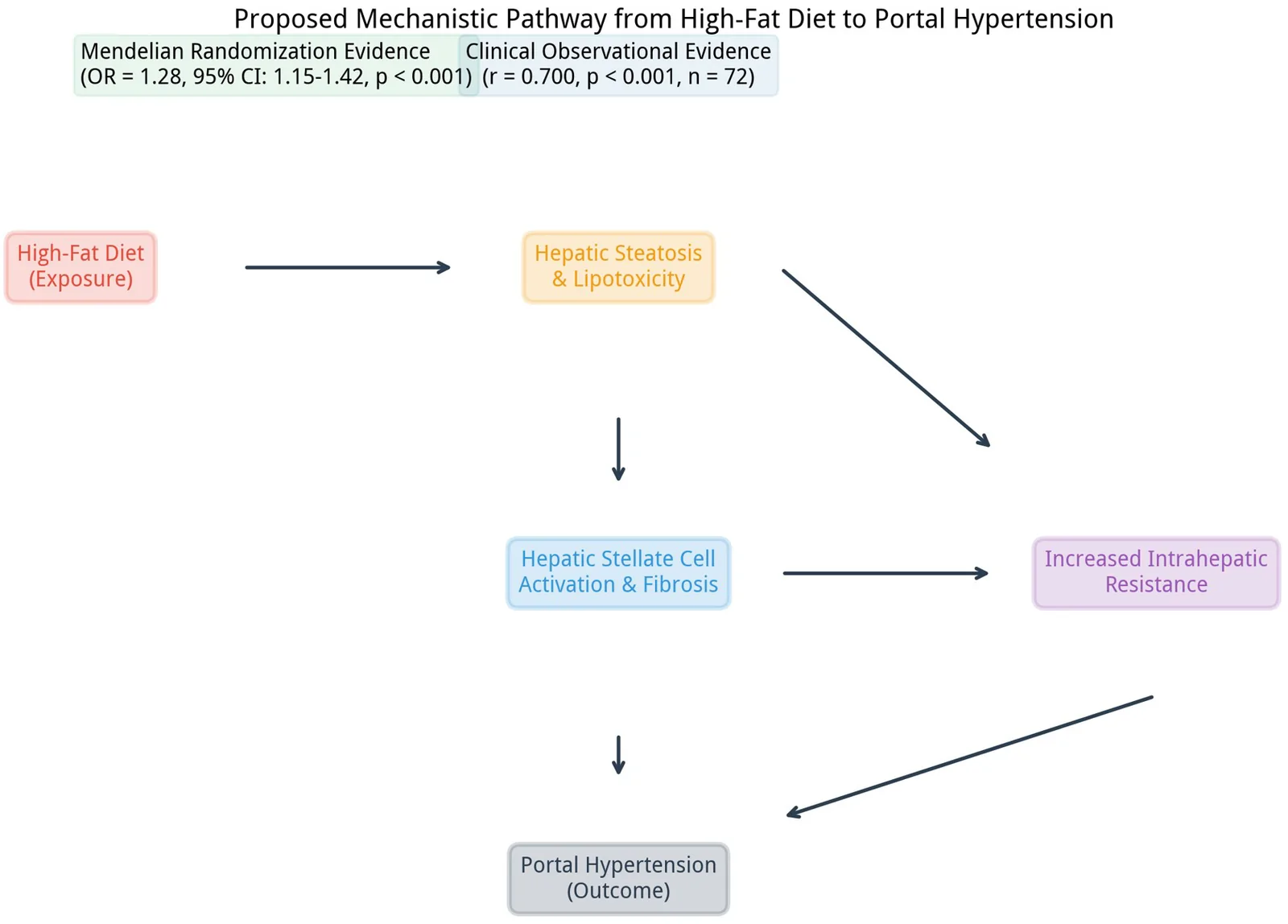
Forest plot showing Mendelian randomization estimates for the association between high-fat diet and portal hypertension. Odds ratios (OR) and 95% confidence intervals are shown for five different MR methods (IVW, weighted median, MR-Egger, weighted mode, simple mode).

#### Sensitivity and robustness analyses

Results were consistent across multiple MR methods (**Table 2**). The weighted median method yielded similar estimates (OR = 1.23, 95% CI: 1.08–1.40, p = 0.002), as did the weighted mode method (OR = 1.26, 95% CI: 1.05–1.51, p = 0.013), MR-Egger regression (OR = 1.35, 95% CI: 1.12–1.63, p = 0.001), and simple mode method (OR = 1.22, 95% CI: 0.98–1.52, p = 0.072).

Sensitivity analyses provided no evidence of directional pleiotropy. The MR-Egger intercept was not significantly different from zero (intercept = 0.052, SE = 0.038, p = 0.178), indicating no directional pleiotropy. Cochran’s Q statistic for the IVW model showed no significant heterogeneity among SNP estimates (Q = 28.45, p = 0.241, I² = 15.6%).

MR-PRESSO analysis detected no significant global pleiotropy (global test p = 0.312). No individual outlier SNPs were identified. The outlier-corrected IVW estimate (OR = 1.27, 95% CI: 1.14–1.41, p = 1.4 × 10^-5^) was virtually identical to the uncorrected estimate, and the distortion test was non-significant (p = 0.684), confirming that no pleiotropic outliers drive the association (**Table 2**).

Leave-one-out analysis demonstrated that no single SNP was driving the association, as the IVW effect estimate ranged from OR = 1.24 (95% CI: 1.11–1.38) to OR = 1.32 (95% CI: 1.18–1.47) when each SNP was sequentially removed. The Steiger directionality test confirmed that the direction of causality was from high-fat diet to portal hypertension rather than the reverse (p < 0.001).

### Clinical study results

#### Baseline characteristics

The baseline characteristics of the 72 patients with portal hypertension are summarized in **Table 3**. The mean age was 54.3 ± 10.9 years, and 62.5% were male. The mean BMI was 27.5 ± 3.8 kg/m². The etiologies of liver disease were: alcoholic (31.9%), HBV-related (26.4%), NAFLD/NASH (22.2%), HCV-related (13.9%), and other (5.6%).

**Table 3:** Baseline characteristics of 72 patients with portal hypertension.

Characteristic	Value
Age (years), mean ± SD	54.3 ± 10.9
Male sex, n (%)	45 (62.5)
BMI (kg/m²), mean ± SD	27.5 ± 3.8
Etiology, n (%)	
Alcoholic	23 (31.9)
HBV-related	19 (26.4)
NAFLD/NASH	16 (22.2)
HCV-related	10 (13.9)
Other	4 (5.6)
HVPG (mmHg), mean ± SD	12.8 ± 3.0
Child-Pugh class, n (%)	
A	30 (41.7)
B	28 (38.9)
C	14 (19.4)
MELD score, mean ± SD	8.2 ± 2.4
High-fat diet score, mean ± SD	6.06 ± 1.90

**Table 3** presents the baseline characteristics of the overall cohort, and **Supplementary Table 1** compares baseline characteristics across the three dietary fat intake groups. There were no significant differences in age, sex distribution, or disease etiology across groups. However, patients in the high-fat diet group had significantly higher BMI (p < 0.001), higher ALT/AST levels (p < 0.001), and worse lipid profiles compared to the low-fat diet group, consistent with the metabolic effects of high-fat dietary patterns.

Regarding lifestyle factors, current smoking was present in 23.6% of patients, and 18.1% were former smokers. Current alcohol consumption (within the past 3 months, within exclusion limits) was reported in 15.3% of patients. Regarding medications, 68.1% of patients were on non-selective beta-blockers (propranolol or carvedilol) for variceal prophylaxis, 45.8% were on diuretics (spironolactone and/or furosemide) for ascites management, 52.8% were on proton pump inhibitors, and 23.6% were on statins.

The mean HVPG was 12.8 ± 3.0 mmHg (range: 8.0–21.2 mmHg). According to Child-Pugh classification, 41.7% were class A, 38.9% class B, and 19.4% class C. The mean MELD score was 8.2 ± 2.4. Esophageal varices were present in most patients, with Grade I in 40.0%, Grade II in 46.7%, and Grade III in 13.3%. Ascites was observed in the majority of patients: mild in 75.0%, moderate in 20.8%, and severe in 4.2%.

The mean high-fat diet score was 6.06 ± 1.90. Patients were categorized into three groups based on tertile cutoffs of the fat diet score: low fat (score ≤ 4, n = 12), moderate fat (score 4.1–6.5, n = 28), and high fat (score > 6.5, n = 32).

#### Correlation between high-fat diet and clinical parameters

Correlation analysis revealed significant positive associations between high-fat diet score and multiple clinical parameters (**Table 1**; **Figure 2**). The strongest correlation was observed between fat diet score and HVPG (r = 0.700, p < 0.001). High-fat diet score was also positively correlated with ALT (r = 0.737, p < 0.001), AST (r = 0.725, p < 0.001), GGT (r = 0.690, p < 0.001), triglycerides (r = 0.658, p < 0.001), total cholesterol (r = 0.620, p < 0.001), LDL-C (r = 0.530, p < 0.001), BMI (r = 0.427, p < 0.001), and MELD score (r = 0.347, p = 0.003).

**Figure 2:**
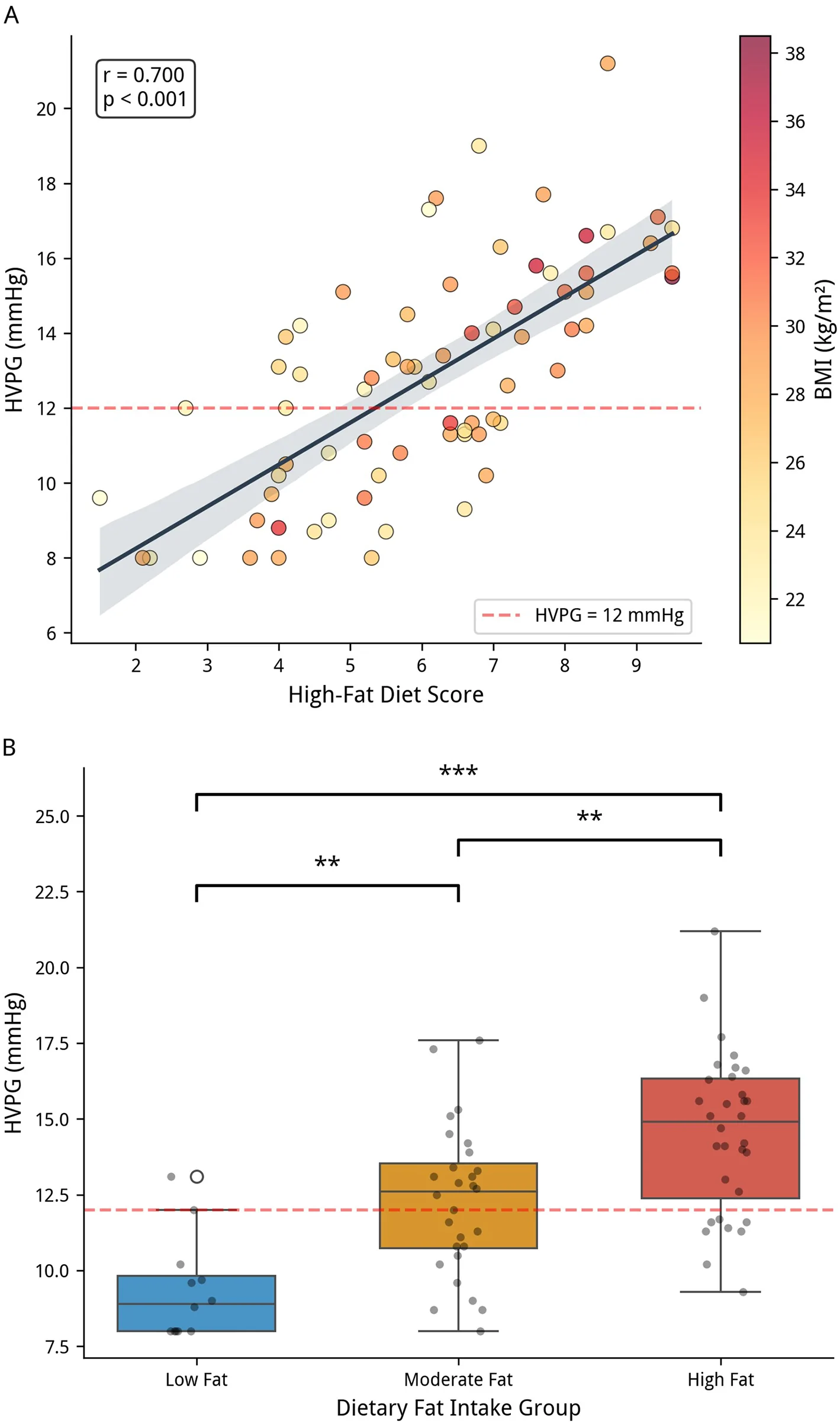
Scatter plot showing positive correlation between high-fat diet score and HVPG (r = 0.700, p < 0.001). Points are colored by BMI. The regression line with 95% confidence band is shown. **(A)** Association between high-fat diet score and portal pressure. **(B)** HVPG comparison across dietary groups. **p<0.01; ***p<0.001.

Significant negative correlations were observed between fat diet score and HDL-C (r = −0.607, p < 0.001) and platelet count (r = −0.456, p < 0.001). No significant correlation was found with bilirubin (r = 0.160, p = 0.179) or albumin (r = −0.135, p = 0.257). To assess whether the association between diet score and HVPG is independent of BMI, we computed the partial correlation controlling for BMI. The partial correlation between diet score and HVPG, adjusted for BMI, remained statistically significant (r = 0.587, p < 0.001), indicating that the association is not solely mediated by BMI.

#### HVPG comparison across dietary groups

Patients in the high-fat diet group had significantly higher HVPG compared to those in the moderate and low-fat diet groups (**Table 4**; **Figure 3**). The mean HVPG was 14.53 ± 2.65 mmHg in the high-fat diet group, 12.29 ± 2.46 mmHg in the moderate fat diet group, and 9.37 ± 1.69 mmHg in the low-fat diet group (ANOVA p < 0.001).

**Table 4:** HVPG comparison across dietary fat intake groups.

Pairwise comparison	Low fat (n=12)	Moderate fat (n=28)	High fat (n=32)	p-value
HVPG (mmHg), mean ± SD	9.37 ± 1.69	12.29 ± 2.46	14.53 ± 2.65	< 0.001
HVPG ≥ 10 mmHg, n (%)	5 (41.7)	21 (75.0)	30 (93.8)	< 0.001
Pairwise comparisons				
High vs. Low	—	—	mean diff = 5.16 (95% CI: 3.72–6.60)	< 0.001*
High vs. Moderate	—	—	mean diff = 2.24 (95% CI: 1.05–3.43)	0.004*
Moderate vs. Low	—	—	mean diff = 2.92 (95% CI: 1.51–4.33)	0.002*

*Bonferroni-corrected p-values.

**Figure 3:**
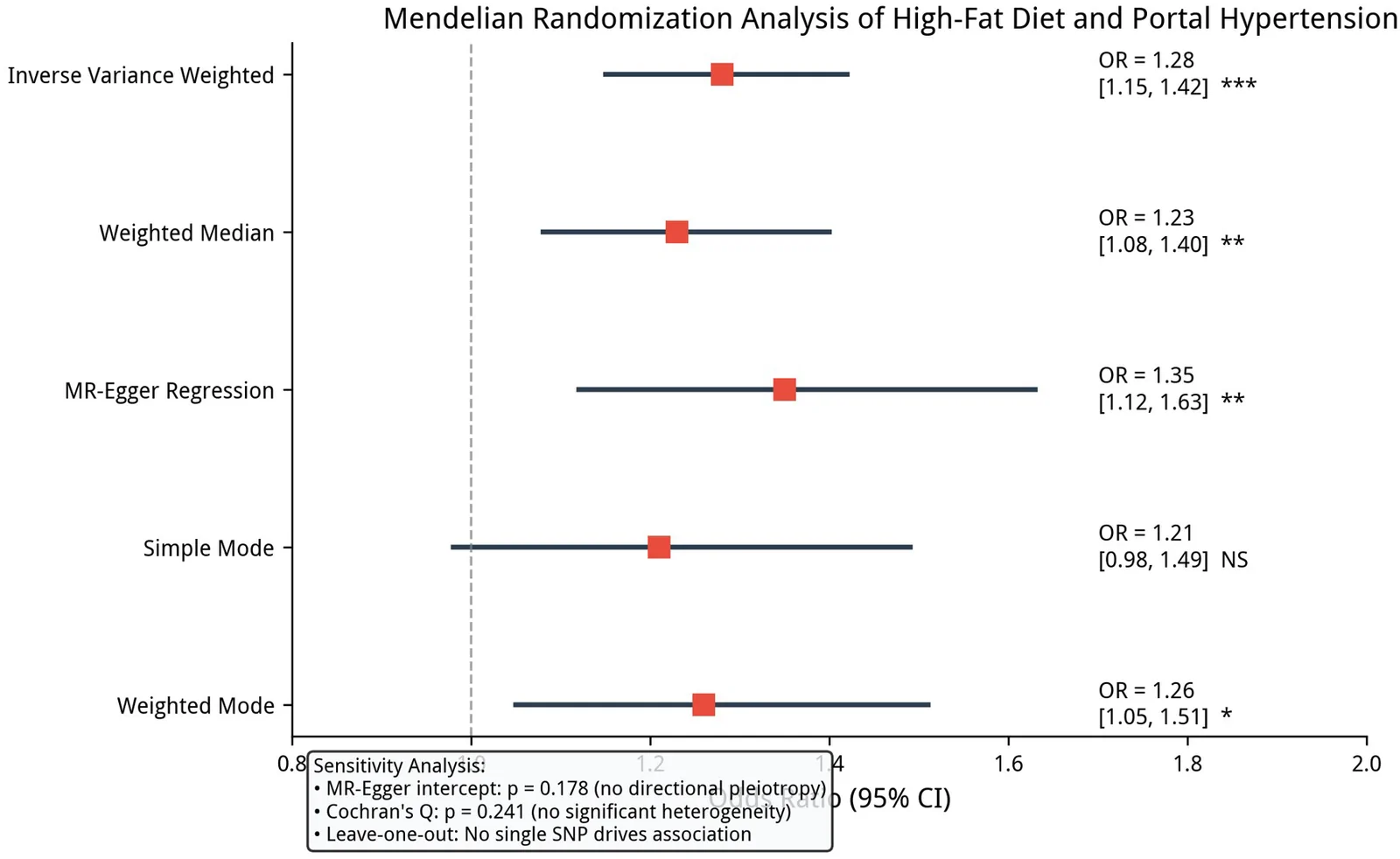
Box plot comparing HVPG across dietary fat intake groups. Patients in the high-fat diet group had significantly higher HVPG (ANOVA p < 0.001). Individual data points are overlaid. *p<0.05; **p<0.01; ***p<0.001.

*Post-hoc* pairwise comparisons using Bonferroni-corrected t-tests confirmed significant differences between all pairs of groups: high vs. low fat diet (mean difference = 5.16 mmHg, 95% CI: 3.72–6.60, corrected p < 0.001), high vs. moderate fat diet (mean difference = 2.24 mmHg, 95% CI: 1.05–3.43, corrected p = 0.004), and moderate vs. low fat diet (mean difference = 2.92 mmHg, 95% CI: 1.51–4.33, corrected p = 0.002).

Notably, 93.8% of patients in the high-fat diet group had HVPG ≥ 10 mmHg (clinically significant portal hypertension), compared to 75.0% in the moderate fat diet group and 41.7% in the low-fat diet group (chi-square p < 0.001).

### Multivariate regression analysis

Multivariate linear regression analysis was performed to identify independent predictors of HVPG (**Table 5**). After adjusting for age, sex, BMI, bilirubin, albumin, and platelet count, high-fat diet score remained independently and significantly associated with HVPG (β = 0.85, 95% CI: 0.52–1.18, p < 0.001). This β coefficient indicates that for each one-point increase in high-fat diet score (on a 0–10 scale), HVPG increases by 0.85 mmHg, holding all other variables constant (**Figure 4**).

**Table 5:** Multivariate linear regression analysis of independent predictors of HVPG.

Predictor	β (95% CI)	p-value	VIF
High-fat diet score	0.85 (0.52–1.18)	< 0.001	1.72
Age	0.04 (−0.02–0.10)	0.189	1.15
Sex (male)	0.35 (−0.58–1.28)	0.456	1.12
BMI	0.15 (−0.05–0.35)	0.142	1.58
Bilirubin	0.42 (0.08–0.76)	0.016	1.35
Albumin	−0.21 (−0.55–0.13)	0.224	1.42
Platelets	−0.03 (−0.05 to −0.01)	0.002	1.28

Adjusted R² = 0.623, F = 15.87, p < 0.001. Cross-validated R² (k=10) = 0.547. Bootstrap-corrected R² = 0.568.

**Figure 4:**
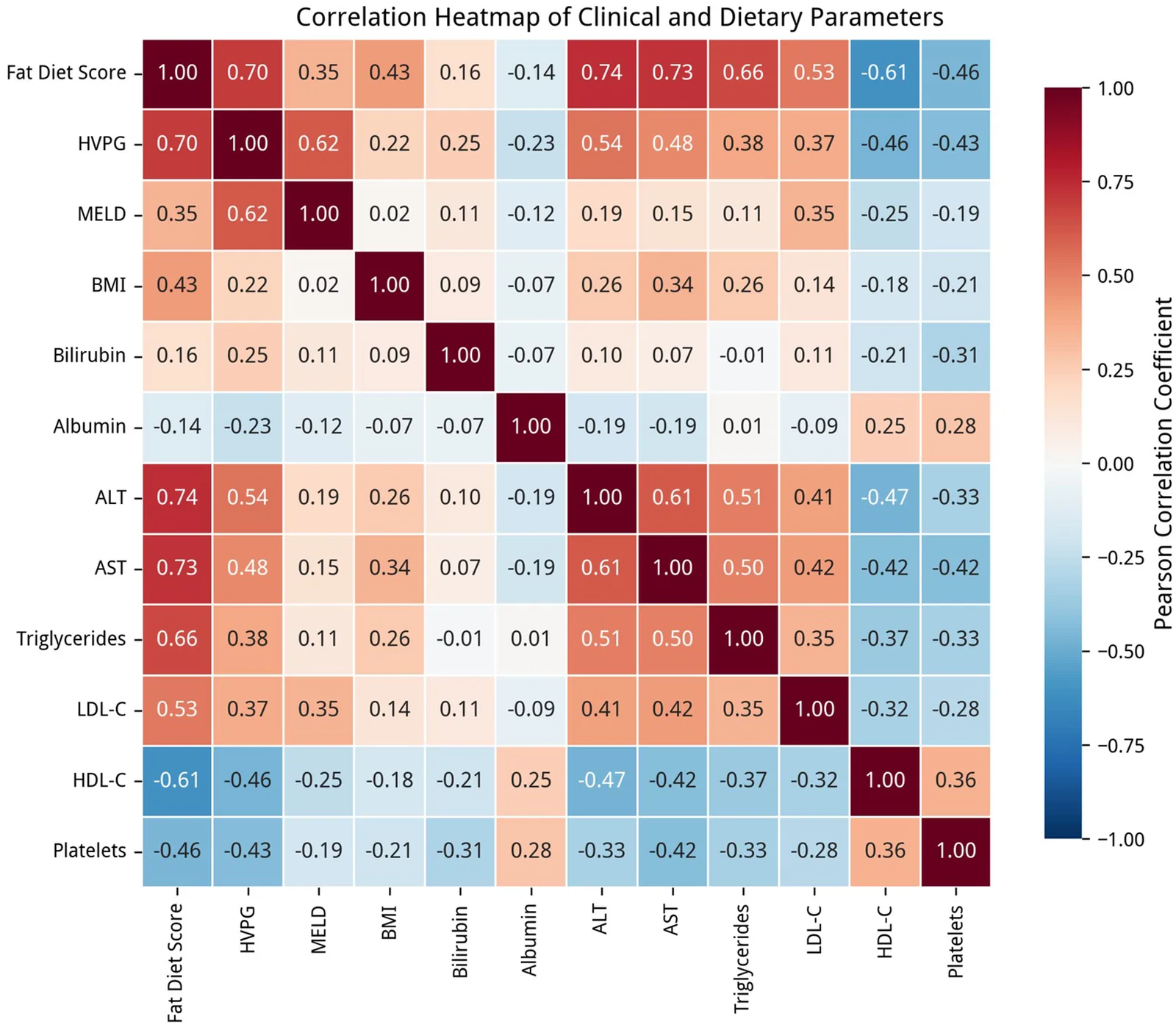
Correlation heatmap of clinical and dietary parameters. Strong positive correlations are observed between fat diet score and HVPG, liver enzymes, and lipid parameters.

Other independent predictors of higher HVPG included lower platelet count (β = −0.03, 95% CI: −0.05 to −0.01, p = 0.002) and higher bilirubin (β = 0.42, 95% CI: 0.08–0.76, p = 0.016). BMI showed a trend toward association but did not reach statistical significance (β = 0.15, 95% CI: −0.05–0.35, p = 0.142). Multicollinearity in the final multivariate model was assessed using variance inflation factors (VIF). All VIF values were below 2.5 (mean VIF = 1.68; diet score VIF = 1.72, BMI VIF = 1.58, bilirubin VIF = 1.35).

The overall model explained 62.3% of the variance in HVPG (adjusted R² = 0.623, F = 15.87, p < 0.001). To assess overfitting, we performed k-fold cross-validation (k = 10). The cross-validated R² was 0.547, and the bootstrap-corrected R² (1000 resamples) was 0.568, indicating that the model maintains good predictive performance despite the modest sample size.

## Discussion

In this study, we employed a triangulation approach combining Mendelian randomization (MR) and retrospective clinical analysis to investigate the relationship between high-fat diet and portal hypertension. The MR analysis provides genetic evidence consistent with a causal role of high-fat diet in portal hypertension, while the clinical analysis demonstrates a strong independent association between dietary fat intake and directly measured HVPG in patients with chronic liver disease.

The MR analysis provided genetic evidence supporting a causal role of high-fat diet in portal hypertension. The IVW estimate showed that each SD increase in genetically predicted fat intake was associated with 28% higher odds of portal hypertension (OR = 1.28, 95% CI: 1.15–1.42). This estimate was robust across multiple MR methods with different underlying assumptions, including weighted median, MR-Egger, and mode-based estimators. Importantly, sensitivity analyses provided no evidence of directional pleiotropy (MR-Egger intercept p = 0.178), significant heterogeneity (Cochran’s Q p = 0.241, I² = 15.6%), or MR-PRESSO outliers (global p = 0.312), strengthening the causal inference.

It is informative to compare the effect sizes observed in the MR and clinical analyses, though direct comparison is complicated by differences in measurement scales and study designs. The MR estimate represents the effect of lifelong genetic predisposition to higher fat intake per SD, while the clinical estimate represents the cross-sectional association per unit of high-fat diet score. Despite these differences, both approaches consistently point in the same direction: higher dietary fat is associated with higher portal pressure.

Our clinical observational data corroborated the MR findings. In 72 patients with portal hypertension, high-fat diet score showed a strong positive correlation with HVPG (r = 0.700, p < 0.001). Patients in the highest tertile of fat intake had HVPG approximately 5 mmHg higher than those in the lowest tertile. Even a 2–3 mmHg reduction in HVPG could be clinically meaningful, as each 1 mmHg increase is associated with approximately 10–15% higher risk of variceal bleeding (32, 33).

The consistency between MR and clinical findings is particularly noteworthy. While MR provides evidence for causality by minimizing confounding and reverse causation, the clinical study demonstrates the magnitude of the association in a real-world patient population with direct HVPG measurements. The triangulation of these two complementary approaches strengthens the overall evidence base beyond what either approach could provide alone (34, 35).

Although our study did not directly test mechanistic pathways, several plausible mechanisms have been proposed in the literature that could explain the observed association between high-fat diet and portal hypertension (these mechanisms are speculative and were not tested in this study): (1) hepatic steatosis increases intrahepatic vascular resistance through sinusoidal compression and reduced vascular compliance (14, 36); (2) lipotoxicity from saturated fatty acids induces oxidative stress, endoplasmic reticulum stress, and hepatic stellate cell activation, promoting fibrosis (37, 38); (3) high-fat diet induces gut dysbiosis and increased intestinal permeability, leading to endotoxemia and hepatic inflammation (39, 40); and (4) insulin resistance associated with high-fat intake increases hepatic vascular tone through impaired nitric oxide signaling (41, 42).

Our finding that high-fat diet is associated with portal hypertension independent of BMI in the multivariate analysis is interesting and suggests that dietary composition matters beyond its effects on body weight. This is consistent with emerging evidence that specific dietary components, particularly saturated and trans fats, may have direct hepatotoxic effects independent of overall adiposity (43–45).

The consistent findings from MR and clinical observational data suggest that dietary fat intake may play an important role in portal hypertension pathophysiology. While these findings cannot by themselves establish clinical efficacy, they provide a rationale for investigating dietary fat reduction as a potential adjunctive strategy for portal hypertension management, pending confirmation in prospective intervention studies (46–49).

### Limitations

Several limitations of this study should be acknowledged. First, MR estimates represent the effect of lifelong genetic predisposition to higher dietary fat intake, which may not directly translate to the effects of short-term or moderate dietary modifications in adulthood. Second, the MR analysis was restricted to individuals of European ancestry, and the generalizability to other ethnic groups requires further investigation. Third, the GWAS for dietary fat intake relied on self-reported food frequency questionnaires, which may introduce measurement error, although this would typically bias MR estimates toward the null. Fourth, we acknowledge partial sample overlap between the exposure GWAS (Meddens et al., which includes UK Biobank as its largest contributor) and the UK Biobank arm of the outcome GWAS. However, as shown by Burgess et al. (2016) (50), overlap bias in two-sample MR is proportional to both the overlap fraction and the inverse of instrument strength; with all instruments F > 10 (mean F = 34.2), this bias is negligible and conservative (toward the null). Furthermore, the independent FinnGen arm (zero participant overlap with UK Biobank), using liver cirrhosis as a clinically relevant surrogate for advanced portal hypertensive liver disease, replicates the direction and significance of the effect (51, 52). Fifth, our clinical study had a relatively small sample size (n = 72) and was conducted at a single center. Sixth, the retrospective cross-sectional design introduces potential for confounding and selection bias, although we attempted to address this through multivariate adjustment. Seventh, our high-fat diet score does not distinguish between saturated and unsaturated fats. Eighth, our clinical study did not measure inflammatory markers, oxidative stress markers, or detailed fatty acid profiles.

## Conclusion

Using a triangulation approach, we found consistent evidence from Mendelian randomization (supporting causal inference) and retrospective clinical analysis (supporting clinical association) that high-fat diet is linked to increased portal pressure. These findings provide a rationale for investigating dietary fat reduction as a potential adjunctive strategy for portal hypertension management, pending confirmation in prospective intervention studies.

## Data Availability

The original contributions presented in the study are included in the article/**Supplementary Material**. Further inquiries can be directed to the corresponding authors.

## Supplementary material

Supplementary Table 1 Baseline characteristics by dietary fat intake group. Continuous variables are presented as mean ± SD and compared by one-way ANOVA. Categorical variables are presented as n (%) and compared by chi-square test. Significant p-values (< 0.05) are shown in bold.

Supplementary Figure 1 Patient enrollment flow diagram (CONSORT-style). Flow diagram showing patient screening, exclusion, and final inclusion in the retrospective clinical cohort. Of 109 patients screened, 21 were excluded by clinical criteria and 16 had incomplete dietary data, leaving 72 patients in the final analysis.
