# Supplementary material for: High-fat diet and portal hypertension: a triangulation approach combining Mendelian randomization with retrospective clinical analysis
Source: Front Gastroenterol (Lausanne). 2026 Sep 29;5:1927449. doi: 10.3389/fgstr.2026.1927449 (PMC13658083; doi:10.3389/fgstr.2026.1927449)
Supplement: Supplementary file 1 [file DataSheet1.docx]

**Supplementary Materials**

**Supplementary Table 1. Baseline Characteristics by Dietary Fat Intake Group**

| **Characteristic** | **Low Fat (n = 12)** | **Moderate Fat (n = 28)** | **High Fat (n = 32)** | **p-value** |
| --- | --- | --- | --- | --- |
| Age (years) | 54.3 ± 8.1 | 54.3 ± 10.4 | 54.3 ± 9.2 | 1.000 |
| Sex |  |  |  |  |
| Male | 8 (66.7) | 17 (60.7) | 20 (62.5) | 0.938 |
| Female | 4 (33.3) | 11 (39.3) | 12 (37.5) | 0.938 |
| BMI (kg/m²) | 25.6 ± 2.5 | 27.9 ± 3.1 | 29.2 ± 3.6 | 0.007 |
| Etiology |  |  |  |  |
| Alcoholic | 3 (25.0) | 9 (32.1) | 11 (34.4) | 0.838 |
| HBV-related | 3 (25.0) | 7 (25.0) | 8 (25.0) | 1.000 |
| NAFLD/NASH | 3 (25.0) | 6 (21.4) | 7 (21.9) | 0.968 |
| HCV-related | 2 (16.7) | 4 (14.3) | 4 (12.5) | 0.936 |
| Other | 1 (8.3) | 2 (7.1) | 2 (6.2) | 0.970 |
| Child-Pugh class |  |  |  |  |
| A | 6 (50.0) | 12 (42.9) | 13 (40.6) | 0.855 |
| B | 5 (41.7) | 13 (46.4) | 16 (50.0) | 0.880 |
| C | 1 (8.3) | 3 (10.7) | 3 (9.4) | 0.969 |
| MELD score | 8.2 ± 2.4 | 8.8 ± 1.5 | 8.1 ± 2.1 | 0.350 |
| HVPG (mmHg) | 9.8 ± 1.5 | 13.3 ± 2.2 | 14.7 ± 3.4 | < 0.001 |
| ALT (U/L) | 45.4 ± 11.9 | 61.4 ± 19.9 | 81.7 ± 26.9 | < 0.001 |
| AST (U/L) | 37.0 ± 14.0 | 47.9 ± 18.0 | 70.0 ± 25.0 | < 0.001 |
| Total cholesterol (mmol/L) | 4.01 ± 0.74 | 4.94 ± 0.63 | 5.48 ± 0.87 | < 0.001 |
| LDL-C (mmol/L) | 2.26 ± 0.56 | 2.87 ± 0.71 | 3.40 ± 0.71 | < 0.001 |
| HDL-C (mmol/L) | 1.39 ± 0.20 | 1.16 ± 0.23 | 1.03 ± 0.22 | < 0.001 |
| Triglycerides (mmol/L) | 1.38 ± 0.45 | 1.60 ± 0.40 | 2.43 ± 0.62 | < 0.001 |
| Smoking status |  |  |  |  |
| Never | 4 (33.3) | 14 (50.0) | 18 (56.2) | 0.400 |
| Current | 4 (33.3) | 6 (21.4) | 7 (21.9) | 0.685 |
| Former | 4 (33.3) | 8 (28.6) | 7 (21.9) | 0.704 |
| Current alcohol consumption |  |  |  |  |
| Yes | 4 (33.3) | 6 (21.4) | 6 (18.8) | 0.580 |
| No | 8 (66.7) | 22 (78.6) | 26 (81.2) | 0.580 |
| Non-selective beta-blockers |  |  |  |  |
| Yes | 4 (33.3) | 17 (60.7) | 20 (62.5) | 0.193 |
| No | 8 (66.7) | 11 (39.3) | 12 (37.5) | 0.193 |
| Diuretics |  |  |  |  |
| Yes | 5 (41.7) | 14 (50.0) | 10 (31.2) | 0.334 |
| No | 7 (58.3) | 14 (50.0) | 22 (68.8) | 0.334 |
| Proton pump inhibitors |  |  |  |  |
| Yes | 6 (50.0) | 13 (46.4) | 15 (46.9) | 0.977 |
| No | 6 (50.0) | 15 (53.6) | 17 (53.1) | 0.977 |
| Statins |  |  |  |  |
| Yes | 3 (25.0) | 5 (17.9) | 9 (28.1) | 0.641 |
| No | 9 (75.0) | 23 (82.1) | 23 (71.9) | 0.641 |

*Continuous variables are presented as mean ± SD and compared by one-way ANOVA. Categorical variables are presented as n (%) and compared by chi-square test. Significant p-values (< 0.05) are shown in bold.*

**Supplementary Figure 1. Patient Enrollment Flow Diagram (CONSORT-style)**

*Flow diagram showing patient screening, exclusion, and final inclusion in the retrospective clinical cohort. Of 109 patients screened, 21 were excluded by clinical criteria and 16 had incomplete dietary data, leaving 72 patients in the final analysis.*
